# Supplementary material for: Incidence and Risk Factors of COVID-19-Associated Pulmonary Aspergillosis in Intensive Care Unit—A Monocentric Retrospective Observational Study
Source: Pathogens. 2021 Oct 22;10(11):1370. doi: 10.3390/pathogens10111370 (PMC8623919; doi:10.3390/pathogens10111370)
Supplement: Supplementary file 1 [file pathogens-10-01370-s001.zip › Supplementary material - Figure S1_Kaplan-Meier Survival curbs of COVID-19 patients in ICU during the 1st wave vs the 2nd wave.pdf]

Figure S1. Kaplan-Meier Survival curbs of COVID-19 patients in ICU during the 1st wave vs the 2nd wave, since hospitalization (a) and since ICU admission (b).

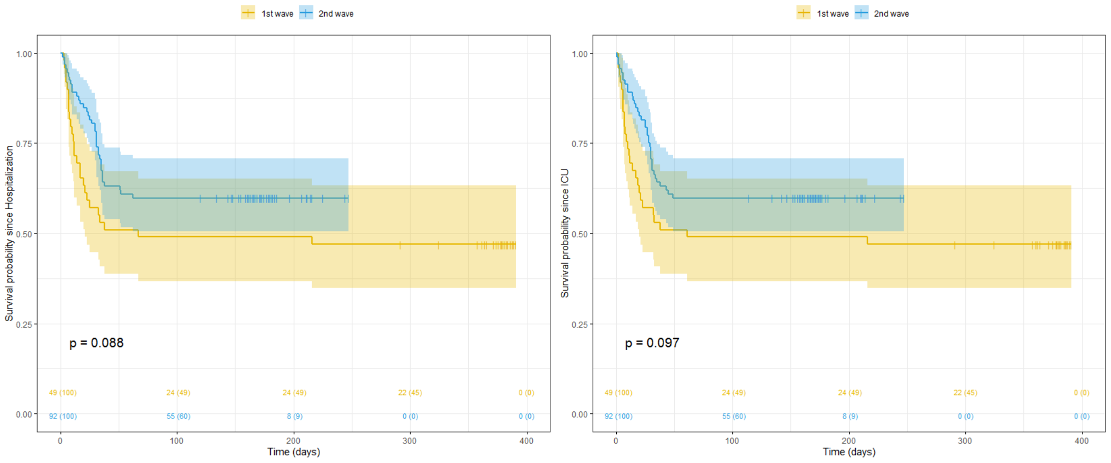

a) **b)**
