# Supplementary material for: Incidence and Risk Factors of COVID-19-Associated Pulmonary Aspergillosis in Intensive Care Unit—A Monocentric Retrospective Observational Study
Source: Pathogens. 2021 Oct 22;10(11):1370. doi: 10.3390/pathogens10111370 (PMC8623919; doi:10.3390/pathogens10111370)
Supplement: Supplementary file 1 [file pathogens-10-01370-s001.zip › Supplementary material - Table S1_Demographic characteristic and comorbidities of the population.pdf]

Table S1. Demographic characteristic and comorbidities of the population

Variable	Categories	N	N (%)	Mean	SD	Min	Q1	Med	Q3	Max
Age (years)		141		62.2	12.5	18.0	56.0	64.0	70.0	87.0
BMI (kg/m²)		120		30.4	6.1	21.2	26.1	29.0	33.0	57.8
	[20; 25[		17 (14.2)							
	[25; 30[		48 (40.0)							
	[30; 35[		36 (30.0)							
	[35; 40[		13 (10.8)							
	≥ 40		6 (5.0)							
Sex		141								
	Men		102 (72.3)							
	Women		39 (27.7)							
Wave		141								
	1st wave		49 (34.7)							
	2nd wave		92 (65.3)							
Hypertension		141	77 (54.6)							
Cerebrovascular disease		141	8 (5.7)							
Diabetes		141	50 (35.5)							
Thrombo-embolic disease		141	6 (4.3)							
COPD		141	16 (11.4)							
Former TB		141	2 (1.4)							
Former Aspergillosis		141	1 (0.7)							
HIV		141	2 (1.4)							
Obesity		120								
	Normal (BMI < 25)		17 (14.2)							
	Overweight (25 ≤ BMI < 30)		48 (40.0)							
	Obesity (BMI ≥ 30)		55 (45.8)							
Cardiac disease		141								
	No		102 (72.3)							
	Coronary heart disease		17 (12.1)							
	Non-coronary heart failure		1 (0.7)							
	AF/Flutter		9 (6.4)							
	Malignant arrhythmia		1 (0.7)							
	Hypertensive cardiopathy		2 (1.4)							
	Prosthetic valve		1 (0.7)							
	Other		3 (2.1)							
	Several		5 (3.6)							
Smoking		121								
	No		71 (58.7)							
	Former		41 (33.9)							
	Active		9 (7.4)							
Alcoholism		121								
	No		109 (90.1)							
	Former		7 (5.8)							
	Active		5 (4.1)							
Lung disease other than COPD		141								
	No		109 (77.3)							
	Asthma		12 (8.5)							
	OSAHS		15 (10.6)							
	Sarcoidosis		1 (0.7)							
	Vasculitis		1 (0.7)							

Variable	Categories	N	N (%)	Mean	SD	Min	Q1	Med	Q3	Max
	PHT		1 (0.7)							
	Asbestosis		2 (1.4)							
CKD		141								
	No (GFR > 60)		130 (92.2)							
	Yes, without ERP		10 (7.1)							
	Haemodialysis		1 (0.7)							
Liver disease		141								
	No		129 (91.5)							
	Cirrhosis		2 (1.4)							
	Hepatitis B		3 (2.1)							
	Hepatitis C		2 (1.4)							
	Other hepatitis		5 (3.6)							
Neoplasia		141								
	No		130 (92.2)							
	Former (> 5 years)		3 (2.1)							
	Recent (< 5 years)		6 (4.3)							
	Active		1 (0.7)							
	Other		1 (0.7)							
Malignant hemopathy		141								
	No		136 (96.5)							
	Lymphoma		3 (2.1)							
	Multiple myeloma		2 (1.4)							
Benign hemopathy		141								
	No		138 (97.9)							
	MGUS		2 (1.4)							
	Thalassemia		1 (0.7)							
Auto-immune disease		141								
	None		129 (91.5)							
	RA		2 (1.4)							
	PMR		1 (0.7)							
	ANCA vasculitis		1 (0.7)							
	Connectivitis		1 (0.7)							
	BID		2 (1.4)							
	Thyroiditis		1 (0.7)							
	Sarcoidosis		1 (0.7)							
	ILD		1 (0.7)							
	Skin disease		1 (0.7)							
	ITP		1 (0.7)							
Immunodeficiency		141								
	No		139 (98.6)							
	Splenectomy		1 (0.7)							
	AIDS		1 (0.7)							
Immunosuppressive treatment		141								
	No		132 (93.6)							
	Chronic corticosteroid therapy (EORTC)		1 (0.7)							
	Chronic corticosteroid therapy (non-EORTC)		3 (2.1)							
	Rituximab		1 (0.7)							
	Mycophenolate mofetil		1 (0.7)							
	Methotrexate		2 (1.4)							
	Combination		1 (0.7)							

AIDS = acquired immunodeficiency syndrome; AF = atrial fibrillation; ANCA = antineutrophil cytoplasm antibody; BID = bowel inflammatory disease; BMI = body mass index; CKD = chronic kidney disease; COPD = chronic obstructive pulmonary disease;

EORTC = European Organization for Research and Treatment of Cancer; ERP = extrarenal purification; GFR = glomerular filtration rate; HIV = human immunodeficiency virus; ILD = interstitial lung disease; ITP = idiopathic thrombocytopenic purpura; Med = median; MGUS = monoclonal gammopathy of unknown signification; OSAHS = obstructive sleep apnea-hypopnoea syndrome; PHT = pulmonary hypertension; PMR = polymyalgia rheumatica; Q1 = first quartile; Q3 = third quartile; RA = rheumatoid arthritis; SD = standard deviation; TB = tuberculosis
