# Supplementary material for: Incidence and Risk Factors of COVID-19-Associated Pulmonary Aspergillosis in Intensive Care Unit—A Monocentric Retrospective Observational Study
Source: Pathogens. 2021 Oct 22;10(11):1370. doi: 10.3390/pathogens10111370 (PMC8623919; doi:10.3390/pathogens10111370)
Supplement: Supplementary file 1 [file pathogens-10-01370-s001.zip › Supplementary material - Table S2_Respiratory support and COVID-19 treatment administered.pdf]

Table S2. Respiratory support and COVID-19 treatment administered.

Variable	Categories	N	N (%)	Mean	SD	Min	Q1	Med	Q3	Max
**Respiratory support**		141								
	Oxygen prongs/mask		35 (24.8)							
	NIV		5 (3.6)							
	MV		88 (62.4)							
	VV ECMO		11 (7.8)							
	AV ECMO		2 (1.4)							
**COVID-19 treatment**										
Antibiotics at admission		140								
	No		23 (16.4)							
	Amoxicillin/clavulanic acid		106 (75.7)							
	Other		11 (7.9)							
AZT and HCQ		140								
	No		91 (65.0)							
	AZT		3 (2.1)							
	HCQ		9 (6.4)							
	AZT + HCQ		37 (26.4)							
Remdesivir		141	5 (3.6)							
DXM (and duration in days)		141	91 (64.5)	8.6	1.8	0.0	9.0	9.0	9.0	11.0
Tocilizumab		141	2 (1.4)							
Siltuximab		141	1 (0.7)							
Anakinra		141	4 (2.8)							
Plasma		141	6 (4.3)							
Other corticosteroids		141	3 (2.1)							

AV = arteriovenous; AZT = azithromycin; DXM = dexamethasone; ECMO = extracorporeal membrane oxygenation; HCQ = hydroxychloroquine; Med = median; MV = mechanical ventilation; NIV = non-invasive ventilation; Q1 = first quartile; Q3 = third quartile; SD = Standard deviation; VV = venovenous
