# Supplementary material for: Incidence and Risk Factors of COVID-19-Associated Pulmonary Aspergillosis in Intensive Care Unit—A Monocentric Retrospective Observational Study
Source: Pathogens. 2021 Oct 22;10(11):1370. doi: 10.3390/pathogens10111370 (PMC8623919; doi:10.3390/pathogens10111370)
Supplement: Supplementary file 1 [file pathogens-10-01370-s001.zip › Supplementary material - Table S3_Risk factors of CAPA - univariate analysis.pdf]

Table S3. Risk factors of CAPA - univariate analysis

			Univariate		Multivariate		
**Variable**	**Categories**	**OR**	**95% CI**	**p-value**	**OR**	**95% CI**	**p-value**
Demographical variables							
Age (years)		1.05	0.98-1.12	0.14			
BMI (kg/m²)		0.97	0.86-1.10	0.66			
Sex	Women	1.33	0.32-5.62	0.69			
Wave	2nd wave	11.3	0.62-203	0.10	>999	2.89->999	0.021
Comorbidities							
Hypertension	Yes	7.30	0.89-60.1	0.064	5.10	0.70-37.3	0.11
Cerebrovascular disease	Yes	6.00	1.02-35.3	0.048	9.98	0.88-114	0.064
Diabetes	Yes	1.50	0.38-5.84	0.56			
Thrombo-embolic disease	Yes	1.02	0.04-24.6	0.99			
COPD	Yes	2.41	0.46-12.7	0.30			
Former TB	Yes	2.75	0.06-120	0.60			
Former Aspergillosis	Yes	4.68	0.05-447	0.51			
HIV	Yes	2.75	0.06-120	0.60			
Obesity	Yes	0.3	0.06-1.57	0.16			
Cardiopathy	Yes	0.73	0.15-3.69	0.71			
Smoking				0.92			
	Former vs Non	0.53	0.02-11.8				
	Active vs Non	0.92	0.23-3.62				
Alcoholism				0.54			
	Former vs Non	1.24	0.05-32.3				
	Active vs Non	3.15	0.41-24.2				
Other pneumopathy	Yes	0.97	0.19-4.93	0.97			
CKD	Yes	1.53	0.17-13.4	0.70			
Hepatopathy	Yes	0.51	0.03-10.4	0.66			
Neoplasia	Yes	0.56	0.03-11.5	0.70			
Malignant hemopathy	Yes	4.00	0.40-40.1	0.24			
Benign hemopathy	Yes	8.13	0.66-99.4	0.10			
Auto-immune disease	Yes	1.38	0.16-12.0	0.77			
Immunodeficiency	Yes	2.75	0.06-120	0.60			
Immunosuppressive treatment	Yes	5.10	0.89-29.2	0.067	0.35	0.04-3.51	0.37
Treatment							
Respiratory support	MV vs Other	12.8	0.71-230	0.084	4.49	0.32-63.0	0.27
Antibiotic at admission	Yes	4.12	0.22-77.8	0.35			
AZT/ HCQ	AZT and/or HCQ	0.088	0.01-1.58	0.099	94.9	0.55->999	0.084
Remdesivir	Yes	1.22	0.05-31.1	0.90			
Dexamethasone	Yes	11.6	0.64-210	0.097	0.41	0.002-103	0.75
Tocilizumab	Yes	2.75	0.06-120	0.60			
Siltuximab	Yes	4.68	0.05-447	0.51			
Anakinra	Yes	1.50	0.05-42.1	0.81			
Convalescent plasma	Yes	3.18	0.33-30.5	0.32			
Other corticosteroids	Yes	1.95	0.06-63.5	0.71			
Antibiotic during hospitalization	At least one	0.82	0.03-20.9	0.90			

AZT = azithromycin; CAPA = COVID-19-associated pulmonary aspergillosis; CI = confidence interval; CKD = chronic kidney disease; COPD = chronic obstructive pulmonary disease; DXM = dexamethasone; HIV = human immunodeficiency virus; MV = mechanical ventilation; NA = not assessed; OR = odd ratio; TB = tuberculosis
