# Supplementary material for: Incidence and Risk Factors of COVID-19-Associated Pulmonary Aspergillosis in Intensive Care Unit—A Monocentric Retrospective Observational Study
Source: Pathogens. 2021 Oct 22;10(11):1370. doi: 10.3390/pathogens10111370 (PMC8623919; doi:10.3390/pathogens10111370)
Supplement: Supplementary file 1 [file pathogens-10-01370-s001.zip › Supplementary material - Table S4_ Comparison of mortality in COVID-19 patients in ICU between the 1st wave and the 2nd wave.pdf]

Table S4. Comparison of mortality in COVID-19 patients in ICU between the 1st wave and the 2nd wave.

Wave	Mortality	Survival since ICU admission	
		P25 (days + 95%CI)	p-value
			0.097
1st wave	26/49 (53.1%)	10.0 (6.0-20.0)	
2nd wave	37/92 (40.2%)	28.5 (19.0-33.0)	

CI = confidence interval; ICU = intensive care unit
