# Supplementary material for: Incidence and Risk Factors of COVID-19-Associated Pulmonary Aspergillosis in Intensive Care Unit—A Monocentric Retrospective Observational Study
Source: Pathogens. 2021 Oct 22;10(11):1370. doi: 10.3390/pathogens10111370 (PMC8623919; doi:10.3390/pathogens10111370)
Supplement: Supplementary file 1 [file pathogens-10-01370-s001.zip › Supplementary material - Tables S1_2_3_4 - Figure S1.pdf]

Table S1. Demographic characteristic and comorbidities of the population

Variable	Categories	N	N (%)	Mean	SD	Min	Q1	Med	Q3	Max
Age (years)		141		62.2	12.5	18.0	56.0	64.0	70.0	87.0
BMI (kg/m²)		120		30.4	6.1	21.2	26.1	29.0	33.0	57.8
	[20; 25[		17 (14.2)							
	[25; 30[		48 (40.0)							
	[30; 35[		36 (30.0)							
	[35; 40[		13 (10.8)							
	≥ 40		6 (5.0)							
Sex		141								
	Men		102 (72.3)							
	Women		39 (27.7)							
Wave		141								
	1st wave		49 (34.7)							
	2nd wave		92 (65.3)							
Hypertension		141	77 (54.6)							
Cerebrovascular disease		141	8 (5.7)							
Diabetes		141	50 (35.5)							
Thrombo-embolic disease		141	6 (4.3)							
COPD		141	16 (11.4)							
Former TB		141	2 (1.4)							
Former Aspergillosis		141	1 (0.7)							
HIV		141	2 (1.4)							
Obesity		120								
	Normal (BMI < 25)		17 (14.2)							
	Overweight (25 ≤ BMI < 30)		48 (40.0)							
	Obesity (BMI ≥ 30)		55 (45.8)							
Cardiac disease		141								
	No		102 (72.3)							
	Coronary heart disease		17 (12.1)							
	Non-coronary heart failure		1 (0.7)							
	AF/Flutter		9 (6.4)							
	Malignant arrhythmia		1 (0.7)							
	Hypertensive cardiopathy		2 (1.4)							
	Prosthetic valve		1 (0.7)							
	Other		3 (2.1)							
	Several		5 (3.6)							
Smoking		121								
	No		71 (58.7)							
	Former		41 (33.9)							
	Active		9 (7.4)							
Alcoholism		121								
	No		109 (90.1)							
	Former		7 (5.8)							
	Active		5 (4.1)							
Lung disease other than COPD		141								
	No		109 (77.3)							
	Asthma		12 (8.5)							
	OSAHS		15 (10.6)							
	Sarcoidosis		1 (0.7)							
	Vasculitis		1 (0.7)							

Variable	Categories	N	N (%)	Mean	SD	Min	Q1	Med	Q3	Max
	PHT		1 (0.7)							
	Asbestosis		2 (1.4)							
CKD		141								
	No (GFR > 60)		130 (92.2)							
	Yes, without ERP		10 (7.1)							
	Haemodialysis		1 (0.7)							
Liver disease		141								
	No		129 (91.5)							
	Cirrhosis		2 (1.4)							
	Hepatitis B		3 (2.1)							
	Hepatitis C		2 (1.4)							
	Other hepatitis		5 (3.6)							
Neoplasia		141								
	No		130 (92.2)							
	Former (> 5 years)		3 (2.1)							
	Recent (< 5 years)		6 (4.3)							
	Active		1 (0.7)							
	Other		1 (0.7)							
Malignant hemopathy		141								
	No		136 (96.5)							
	Lymphoma		3 (2.1)							
	Multiple myeloma		2 (1.4)							
Benign hemopathy		141								
	No		138 (97.9)							
	MGUS		2 (1.4)							
	Thalassemia		1 (0.7)							
Auto-immune disease		141								
	None		129 (91.5)							
	RA		2 (1.4)							
	PMR		1 (0.7)							
	ANCA vasculitis		1 (0.7)							
	Connectivitis		1 (0.7)							
	BID		2 (1.4)							
	Thyroiditis		1 (0.7)							
	Sarcoidosis		1 (0.7)							
	ILD		1 (0.7)							
	Skin disease		1 (0.7)							
	ITP		1 (0.7)							
Immunodeficiency		141								
	No		139 (98.6)							
	Splenectomy		1 (0.7)							
	AIDS		1 (0.7)							
Immunosuppressive treatment		141								
	No		132 (93.6)							
	Chronic corticosteroid therapy (EORTC)		1 (0.7)							
	Chronic corticosteroid therapy (non-EORTC)		3 (2.1)							
	Rituximab		1 (0.7)							
	Mycophenolate mofetil		1 (0.7)							
	Methotrexate		2 (1.4)							
	Combination		1 (0.7)							

AIDS = acquired immunodeficiency syndrome; AF = atrial fibrillation; ANCA = antineutrophil cytoplasm antibody; BID = bowel inflammatory disease; BMI = body mass index; CKD = chronic kidney disease; COPD = chronic obstructive pulmonary disease;

EORTC = European Organization for Research and Treatment of Cancer; ERP = extrarenal purification; GFR = glomerular filtration rate; HIV = human immunodeficiency virus; ILD = interstitial lung disease; ITP = idiopathic thrombocytopenic purpura; Med = median; MGUS = monoclonal gammopathy of unknown signification; OSAHS = obstructive sleep apnea-hypopnoea syndrome; PHT = pulmonary hypertension; PMR = polymyalgia rheumatica; Q1 = first quartile; Q3 = third quartile; RA = rheumatoid arthritis; SD = standard deviation; TB = tuberculosis;

Table S2. Respiratory support and COVID-19 treatment administered.

Variable	Categories	N	N (%)	Mean	SD	Min	Q1	Med	Q3	Max
**Respiratory support**		141								
	Oxygen prongs/mask		35 (24.8)							
	NIV		5 (3.6)							
	MV		88 (62.4)							
	VV ECMO		11 (7.8)							
	AV ECMO		2 (1.4)							
**COVID-19 treatment**										
Antibiotics at admission		140								
	No		23 (16.4)							
	Amoxicillin/clavulanic acid		106 (75.7)							
	Other		11 (7.9)							
AZT and HCQ		140								
	No		91 (65.0)							
	AZT		3 (2.1)							
	HCQ		9 (6.4)							
	AZT + HCQ		37 (26.4)							
Remdesivir		141	5 (3.6)							
DXM (and duration in days)		141	91 (64.5)	8.6	1.8	0.0	9.0	9.0	9.0	11.0
Tocilizumab		141	2 (1.4)							
Siltuximab		141	1 (0.7)							
Anakinra		141	4 (2.8)							
Plasma		141	6 (4.3)							
Other corticosteroids		141	3 (2.1)							

AV = arteriovenous; AZT = azithromycin; DXM = dexamethasone; ECMO = extracorporeal membrane oxygenation; HCQ = hydroxychloroquine; Med = median; MV = mechanical ventilation; NIV = non-invasive ventilation; Q1 = first quartile; Q3 = third quartile; SD = Standard deviation; VV = venovenous

Table S3. Risk factors of CAPA - univariate analysis

Variable	Category	Non-CAPA (N=132)		CAPA (N=9)		OR	95% CI	p-value
		**N**	**N (%)**	**N**	**N (%)**			
Hypertension		132	69 (89.6)	9	8 (10.4)	7.30	0.89-60.1	0.064
Cerebrovascular disease		132	6 (75.0)	9	2 (25.0)	6.00	1.02-35.3	0.048
Diabetes		132	46 (92.0)	9	4 (8.0)	1.50	0.38-5.84	0.56
Thrombo-embolic disease		132	6 (100.0)	9	0 (0.0)	1.02	0.04-24.6	0.99
COPD		132	14 (87.5)	9	2 (12.5)	2.41	0.46-12.7	0.30
Former TB		132	2 (100.0)	9	0 (0.0)	2.75	0.06-120	0.60
Former Aspergillosis		132	1 (100.0)	9	0 (0.0)	4.68	0.05-447	0.51
HIV		132	2 (100.0)	9	0 (0.0)	2.75	0.06-120	0.60
Obesity		132	53 (96.4)	9	2 (3.6)	0.3	0.06-1.57	0.16
Cardiac disease (no distinction)		132	37 (94.9)	9	2 (5.1)	0.73	0.15-3.69	0.71
Smoking		112		9				0.92
	No		65 (91.6)		6 (8.4)	1.00		
	Former		38 (92.7)		3 (7.3)	0.53	0.02-11.8	
	Active		9 (100.0)		0 (0.0)	0.92	0.23-3.62	
Alcoholism		113		8				0.54
	No		102 (93.6)		7 (6.4)	1.00		
	Former		6 (85.7)		1 (14.3)	1.24	0.05-32.3	
	Active		5 (100.0)		0 (0.0)	3.15	0.41-24.2	
Lung disease other than COPD (no distinction)		132	30 (93.8)	9	2 (6.3)	0.97	0.19-4.93	0.97
CKD (no distinction)		132	10 (90.9)	9	1 (9.1)	1.53	0.17-13.4	0.70
Liver disease (no distinction)		132	12 (100.0)	9	0 (0.0)	0.51	0.03-10.4	0.66
Neoplasia (no distinction)		132	11 (100.0)	9	0 (0.0)	0.56	0.03-11.5	0.70
Malignant hemopathy (no distinction)		132	4 (80.0)	9	1 (20.0)	4.00	0.40-40.1	0.24
Benign hemopathy (no distinction)		132	2 (66.7)	9	1 (33.3)	8.13	0.66-99.4	0.10
Auto-immune disease (no distinction)		132	11 (91.7)	9	1 (8.3)	1.38	0.16-12.0	0.77
Immunodeficiency (no distinction)		132	2 (100.0)	9	0 (0.0)	2.75	0.06-120	0.60
Immunosuppressive treatment (no distinction)		132	7 (77.8)	9	2 (22.2)	5.10	0.89-29.2	0.067
Respiratory support (MV vs other)		132	79 (89.8)	9	9 (10.2)	12.8	0.71-230	0.084
Antibiotic at admission (no distinction)		131	108 (92.3)	9	9 (7.7)	4.12	0.22-77.8	0.35
AZT and/or HCQ		131	49 (100.0)	9	0 (0.0)	0.088	0.01-1.58	0.099
Remdesivir		132	5 (100.0)	9	0 (0.0)	1.22	0.05-31.1	0.90
DXM		132	82 (90.1)	9	9 (9.9)	11.6	0.64-210	0.097
Tocilizumab		132	2 (100.0)	9	0 (0.0)	2.75	0.06-120	0.60
Siltuximab		132	1 (100.0)	9	0 (0.0)	4.68	0.05-447	0.51
Anakinra		132	4 (100.0)	9	0 (0.0)	1.50	0.05-42.1	0.81
Convalescent plasma		132	5 (83.3)	9	1 (16.7)	3.18	0.33-30.5	0.32
Other corticosteroids		132	3 (100.0)	9	0 (0.0)	1.95	0.06-63.5	0.71
Antibiotics during hospitalization		132	127 (93.4)	9	9 (6.6)	0.82	0.03-20.9	0.90

AZT = azithromycin; CAPA = COVID-19-associated pulmonary aspergillosis; CI = confidence interval; CKD = chronic kidney disease; COPD = chronic obstructive pulmonary disease; DXM = dexamethasone; HIV = human immunodeficiency virus; MV = mechanical ventilation; NA = not assessed; OR = odd ratio; TB = tuberculosis

Table S4. Comparison of mortality in COVID-19 patients in ICU between the 1st wave and the 2nd wave.

**Wave**	**Mortality**	**Survival since ICU admission**<br>**P25 (days + 95%CI)**	**p-value**
			0.097
1st wave	26/49 (53.1%)	10.0 (6.0-20.0)	
2nd wave	37/92 (40.2%)	28.5 (19.0-33.0)	

CI = confidence interval; ICU = intensive care unit

Figure S1. Kaplan-Meier Survival curbs of COVID-19 patients in ICU during the 1st wave vs the 2nd wave, since hospitalization (a) and since ICU admission (b).

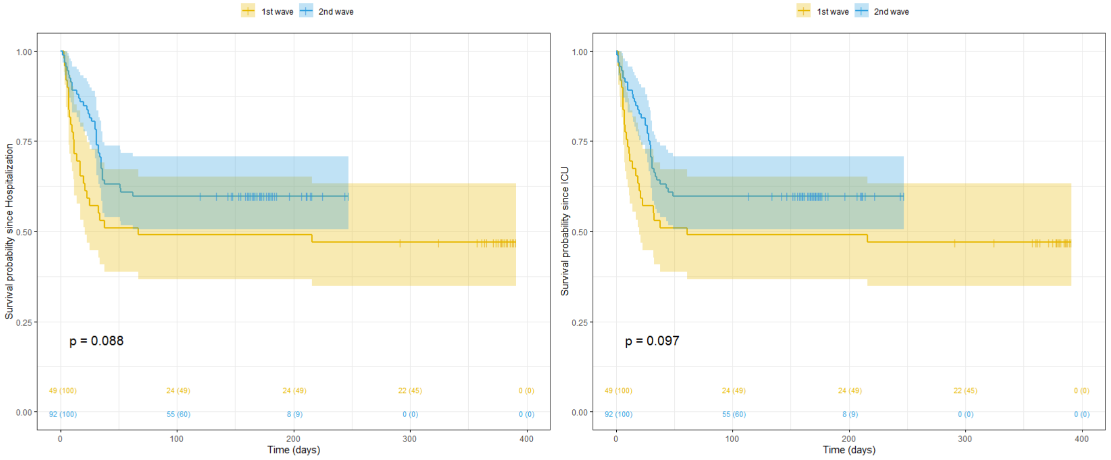

a) **b)**
